# Introducing the society for immunotherapy of cancer’s new journal: journal for immunoTherapy of cancer

**DOI:** 10.1186/2051-1426-1-1

**Published:** 2013-05-29

**Authors:** Pedro J Romero, Tara Withington, Francesco Marincola

**Affiliations:** 1 Ludwig Center for Cancer Research, Lausanne, Switzerland; 2 Society for Immunotherapy of Cancer (SITC), Milwaukee, WI, USA; 3 Sidra Medical and Research Center, Doha, Qatar

## 

The field of cancer immunology and immunotherapy is experiencing a tremendous increase in excitement and momentum, catalyzed by the latest approvals of immunotherapy-based treatments in multiple cancer types. Contributing to the excitement, more cancer vaccine candidates, immunomodulatory monoclonal antibodies and cellular therapies are showing great promise and major pharma companies are driving their development into future novel cancer treatments.

Through extensive research and discussion among both the base and the leadership of the Society for Immunotherapy of Cancer (SITC) in the last several years, it became clear to SITC that there was a need for a targeted and broadly-accessible publication platform dedicated to advancing the science of tumor immunology and cancer immunotherapy. To fulfill this need and enrich communication between the many stakeholders in the tumor immunology and cancer immunotherapy community, SITC has created the *Journal for ImmunoTherapy of Cancer (JITC).* This new journal will serve the cancer immunotherapy community, to be our community’s outlet for the publication of original research articles, literature reviews, opinion/position papers and to be the prime forum to discuss the critical issues in tumor immunology and cancer immunotherapy research. The editorial board was constituted to bring together outstanding leaders in both basic and clinical tumor immunology and cancer immunotherapy fields, and will be focused on processing manuscripts and steering the journal in its initial stages in close collaboration with SITC. The journal is owned by the SITC. It is a joint venture with BioMed Central (BMC) -- a major power house in open access publishing, which organizes 250 peer-reviewed open access journals.

## What do we offer our authors?

First, we strive to publish high quality papers after an efficient peer review process that adheres to the principles of fairness and scientific excellence. We welcome original contributions in the following areas:

•Basic Tumor Immunology – Edited by Cornelis J.M. Melief, ISA Therapeutics BV, The Netherlands. This section will cover innate and adaptive anti-tumor immune mechanisms, immune regulation, immune response, cancer and inflammation, tumor antigens, preclinical models, chemotherapy and radiotherapy interactions with the anti-tumor immune response.

•Clinical/Translational Cancer Immunotherapy – Edited by F. Stephen Hodi, Jr., Dana Farber Cancer Institute, Boston. This section will cover first in man clinical trials, phase II/III clinical studies of immunotherapy, combination therapy, cell therapy, oncolytic viruses; immune monitoring investigations, tumor microenvironment, host genetics and clinical outcome and updates on current clinical trials.

•Immunotherapy Biomarkers – Edited by Lisa H. Butterfield, University of Pittsburgh Cancer Institute. This section will cover predictive and prognostic biomarker studies relating to immunotherapy, including cellular, serologic, tissue and gene expression studies in cancer immunotherapy as well as mechanisms of action.

•Moreover, JITC is happy to publish topical reviews, points of view articles and hypotheses in a section edited by Thomas F. Gajewski, University of Chicago and Bernard A. Fox, Earle A. Chiles Research Institute, Providence Cancer Center, Portland to trigger discussion on hot topics and innovative concepts in the field.

Second, JITC offers immediate wide visibility for the articles appearing online. All papers published in JITC will be followed by alerts reaching our 5,000 researchers spread across institutions worldwide. In addition to this, those articles will also be afforded high exposure across BMC’s websites and to their extensive readership. Thus, authors can be certain that their articles will be immediately visible to the entire cancer immunology and immunotherapy community. This certainly ensures immediate and wide impact with all our peers.

Third, for the first time, our authors will enjoy all the advantages offered by open access publishing with a worldwide outreach. All articles are freely and universally accessible online, so an author’s work is available to readers at no cost and not limited by their library’s budget. This ensures that author’s work is disseminated to the widest possible audience.

Open access journals have the potential to reach a much larger set of readers than any subscription-based journal, in print and online [1]. Some studies have suggested a correlation between open access, higher downloads and higher citations, leading to a higher Impact Factor [2,3]. Authors hold copyright for their work and grant anyone the right to reproduce and disseminate the article, provided that it is correctly cited and no errors are introduced. The journal’s articles are archived in PubMed Central [4], and other freely accessible full-text repositories. This complies with the policies of a number of funding bodies including the Wellcome Trust, NIH and Howard Hughes Medical Institute [5-8]. A country’s economy will not influence its researchers’ ability to access articles because resource-poor countries (and institutions) will be able to read the same material as wealthier ones (although creating access to the internet is another matter [9]).

As part of SITC’s commitment to give the tumor immunology and cancer immunotherapy community a potent tool of scientific and scholar communication, the Society will be completely covering the publishing fees to its members during the inaugural first year of JITC. We of course also welcome nonmember submissions. For the latter, we would like to emphasize that article processing charges cover the cost of the publication process to allow free and immediate access to your research articles. It is a flat fee and there are no additional charges for color figures, movies or large datasets. These charges ensure transparency and allow the publisher to compete to provide the best service and the best price.

We will be working very hard to make JITC the prime venue for publishing the latest results coming from the laboratories and research groups not only of SITC members, but also from the broader cancer immunology community. In addition, we will utilize this outlet as a powerful lever to advance the science and the biomedical applications of tumor immunology. Your Editorial Board is looking forward to reviewing and publishing your papers in the exciting years to come.
